# Designing, implementing, and evaluating the Patient Navigation Model: Filling the gaps in the HIV continuum of care in Los Angeles County

**DOI:** 10.1186/1748-5908-10-S1-A53

**Published:** 2015-08-20

**Authors:** Stella Gukasyan

**Affiliations:** 1 AIDS Project Los Angeles, Los Angeles, CA 90005, USA

## Objective

HIV treatment is only manageable with access to adequate medical care and effective treatment. Gardner et al. [1] developed a cascade of engagement, and retention in HIV care and treatment. The fundamental elements of the continuum of care were confirmed by the Centers for Disease Control's Morbidity and Mortality Weekly Report of December 2, 2011. This report stated that only 19% of HIV-positive individuals have been able to attain viral suppression. Although various programs have utilized patient navigation, there is no evidence-based model that has been evaluated. Our intervention combines trained professionals conducting a minimum of four structured sessions with rigorous evaluation and cost analysis component in partnership with Johns Hopkins Bloomberg School of Public Health.

## Methods

Two hundred thirty five clients, not in medical care and or individuals who have not reached viral suppression have been identified and enrolled. The implementation of this intervention includes emphasis on the importance of follow-up and retention beyond the initial medical appointment, addressing non-HIV medical care, and understanding barriers to attaining viral suppression. A local and national rigorous mixed-methods evaluation is embedded in the thread of the intervention, tracking outcomes at six-month intervals, over an eighteen- month period.

## Results

We have been able to obtain desired changes in health and clinical outcomes for our cohort over an eighteen-month period. Viral suppression has trended from 41% at enrollment to 59% within six months, 71% within 12-months, and 65% after 18-months of enrollment (client racial demographic 24% White, 25% African-American, 43% Latino). At six-months after enrollment, 87% of clients were retained in medical care. By conducting an extensive local and national evaluation, our model clearly highlights and refines best practices for training patient navigators to implement a navigation program to meet the ever-changing needs of HIV-positive individuals.

## Source of Funding

AIDS United, Access2Care Initiative.
